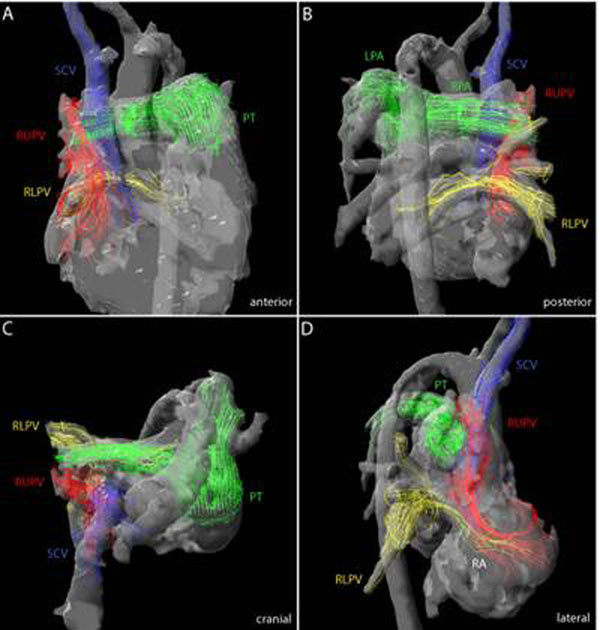# Selective pulmonary venous flow visualization and quantification by flow-sensitive four-dimensional cine magnetic resonance imaging facilitates and improves the accurate diagnosis of partial anomalous pulmonary venous drainage

**DOI:** 10.1186/1532-429X-13-S1-P214

**Published:** 2011-02-02

**Authors:** Sarah Nordmeyer, Felix Berger, Titus Kuehne, Eugénie Riesenkampff

**Affiliations:** 1 Deutsches Herzzentrum Berlin, Berlin, Germany

## Objectives

To assess if flow-sensitive four-dimensional velocity encoded cine magnetic resonance imaging (4D VEC MRI) adds value in diagnosing patients with suspected partial anomalous pulmonary venous drainage (PAPVD).

## Methods

In six patients with echocardiographically suspected PAPVD, anatomy was evaluated using standard magnetic resonance imaging including angiography. Functional analysis included shunt calculations from standard flow-measurements. Furthermore, 4D VEC MRI was used for visualization of maldraining pulmonary veins and quantification of flow via the maldraining veins and interatrial communications, if present.

## Results

In all patients, the diagnosis of PAPVD was confirmed by standard magnetic resonance imaging. Shunt volumes ranged from 1.4:1 to 4.7:1. Drainage sites were the superior caval vein (n=5) or the vertical vein (n=1). Multiple maldraining pulmonary veins were found in three patients. Pulmonary arteries and veins could be clearly distinguished by selective visualization using 4D VEC MRI. Flow measured individually in maldraining pulmonary veins (n=6 patients) and across the interatrial communication (n=3 patients) revealed a percentage of the overall shunt volume of 30 to 100% and 58 to 70%, respectively. Different flow characteristics and directions of maldraining pulmonary veins and across the interatrial communication could be visualized during ventricular systole and diastole.

## Conclusion

Selective visualization of individual vessels and their flow characteristics by 4D VEC MRI facilitates to distinguish adjacent pulmonary arteries and veins and thus improves accurate diagnosis of maldraining pulmonary veins. By detailed quantification of shunt volumes additional information for planning of treatment strategies is provided. This method adds clinical value and might replace contrast-enhanced magnetic resonance angiography in these patients in the future.

Selective visualization of streamlines in a child diagnosed with anomalous pulmonary venous drainage of the right upper pulmonary vein into the superior caval vein (SCV) and presence of an interatrial communication in different perspectives.

Red streamlines represent the flow of the maldraining right upper pulmonary vein (RUPV) entering the right atrium (RA) together with the blue streamlines of the SCV (panel A and B).

The green streamlines in the pulmonary trunk (PT), right and left pulmonary artery (RPA and LPA) allow distinguishing clearly peripheral pulmonary arteries (green) and veins (yellow and red, panel C).

The yellow streamlines of the anatomic correctly draining right lower pulmonary vein (RLPV) merge with the red streamlines representing a left to right shunt across the interatrial communication and thus functional maldrainage during ventricular systole (panel D).

**Figure 1:**